# Chloride of Lime as an Insecticide

**Published:** 1862-07

**Authors:** 


### CHLORIDE OF LIME AS AN INSECTICIDE.

In scattering chloride of lime on a plank in a stable, all kinds of flies, but more especially biting flies, were quickly got rid of. Sprinkling beds of vegetables with even a weak solution of this salt, effectually preserves them from the attacks of caterpillars, butterflies, mordella, slugs, etc. It has the same effect when sprinkled on the foliage of fruit trees. A paste of one part of powdered chloride of lime and one-half part of some fatty matter, placed in a narrow band round the trunk of the tree, prevents insects from creeping up it. It has even been noticed that rats and mice quit places in which a certain quantity of chloride of lime has been spread. This salt, dried and finely powdered, can, no doubt, be employed for the same purposes as flour of sulphur, and be spread by the same means.
—*Chem. News, London, from Dingler's Polytechnisches Journal.*

